# Proposed mechanism for reduced jugular vein flow in microgravity

**DOI:** 10.14814/phy2.14782

**Published:** 2021-05-01

**Authors:** Mimi Lan, Scott D. Phillips, Veronique Archambault‐Leger, Ariane B. Chepko, Rongfei Lu, Allison P. Anderson, Kseniya S. Masterova, Abigail M. Fellows, Ryan J. Halter, Jay C. Buckey

**Affiliations:** ^1^ Thayer School of Engineering at Dartmouth Hanover NH USA; ^2^ Creare LLC Hanover NH USA; ^3^ Stanford University Palo Alto CA USA; ^4^ University of Colorado Boulder Boulder CO USA; ^5^ University of Texas Medical Branch Galveston TX USA; ^6^ Geisel School of Medicine at Dartmouth College Lebanon NH USA

**Keywords:** jugular venous blood flow, microgravity, numerical model, vertebral plexus

## Abstract

Internal jugular flow is reduced in space compared with supine values, which can be associated with internal jugular vein (IJV) thrombosis. The mechanism is unknown but important to understand to prevent potentially serious vein thromboses on long duration flights. We used a novel, microgravity‐focused numerical model of the cranial vascular circulation to develop hypotheses for the reduced flow. This model includes the effects of removing hydrostatic gradients and tissue compressive forces – unique effects of weightlessness. The IJV in the model incorporates sensitivity to transmural pressure across the vein, which can dramatically affect resistance and flow in the vein. The model predicts reduced IJV flow in space. Although tissue weight in the neck is reduced in weightlessness, increasing transmural pressure, this is more than offset by the reduction in venous pressure produced by the loss of hydrostatic gradients and tissue pressures throughout the body. This results in a negative transmural pressure and increased IJV resistance. Unlike the IJV, the walls of the vertebral plexus are rigid; transmural pressure does not affect its resistance and so its flow increases in microgravity. This overall result is supported by spaceflight measurements, showing reduced IJV area inflight compared with supine values preflight. Significantly, this hypothesis suggests that interventions that further decrease internal IJV pressure (such as lower body negative pressure), which are not assisted by other drainage mechanisms (e.g. gravity), might lead to stagnant flow or IJV collapse with reduced flow, which could increase rather than decrease the risk of venous thrombosis.

## Graphical abstract

Internal jugular flow is reduced in space, which can be associated with internal jugular vein (IJV) thrombosis. We used a novel, microgravity‐focused numerical model of the cranial vascular circulation to develop hypotheses for the reduced flow. Weightlessness reduces venous pressures throughout the body which reduces internal jugular vein diameter and thus internal jugular venous flow.

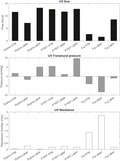

## 1 INTRODUCTION

Blood drains from the brain via two primary pathways: the internal jugular veins (IJV) and the vertebral venous plexus (VP) (Gisolf et al., 2004). On Earth, the IJV drainage pathway predominates supine (66% of blood flow), whereas the VP is the main pathway upright (Doepp et al., 2004). In microgravity, the drainage pathway through the IJV is reduced to varying degrees compared with supine. In some individuals, IJV blood flow has been measured to be stagnant or even to flow backwards (Marshall‐Goebel et al., 2019). This slow or stagnant flow is believed to have led to a jugular vein thrombosis that required inflight treatment (Marshall‐Goebel et al., 2019).

The cause of reduced IJV flow in space is not well understood, but understanding it is important for preventing in‐flight vein thromboses. To explore possible causes, we used a novel numerical model of the cranial circulation. The model was created using MATLAB^®^ Simscape Fluids^™^ (MathWorks^®^, Natick, MA). The model simulates changes in body fluid distribution and pressures in various body positions (supine, prone, head down tilt), different gravity conditions (0‐g, 1‐g, etc.), different body sizes (neck, chest, waist circumference), and the presence of an external pressure device (lower body negative pressure (LBNP), lower body positive pressure (LBPP)) used on the lower extremities.

**FIGURE 1:**
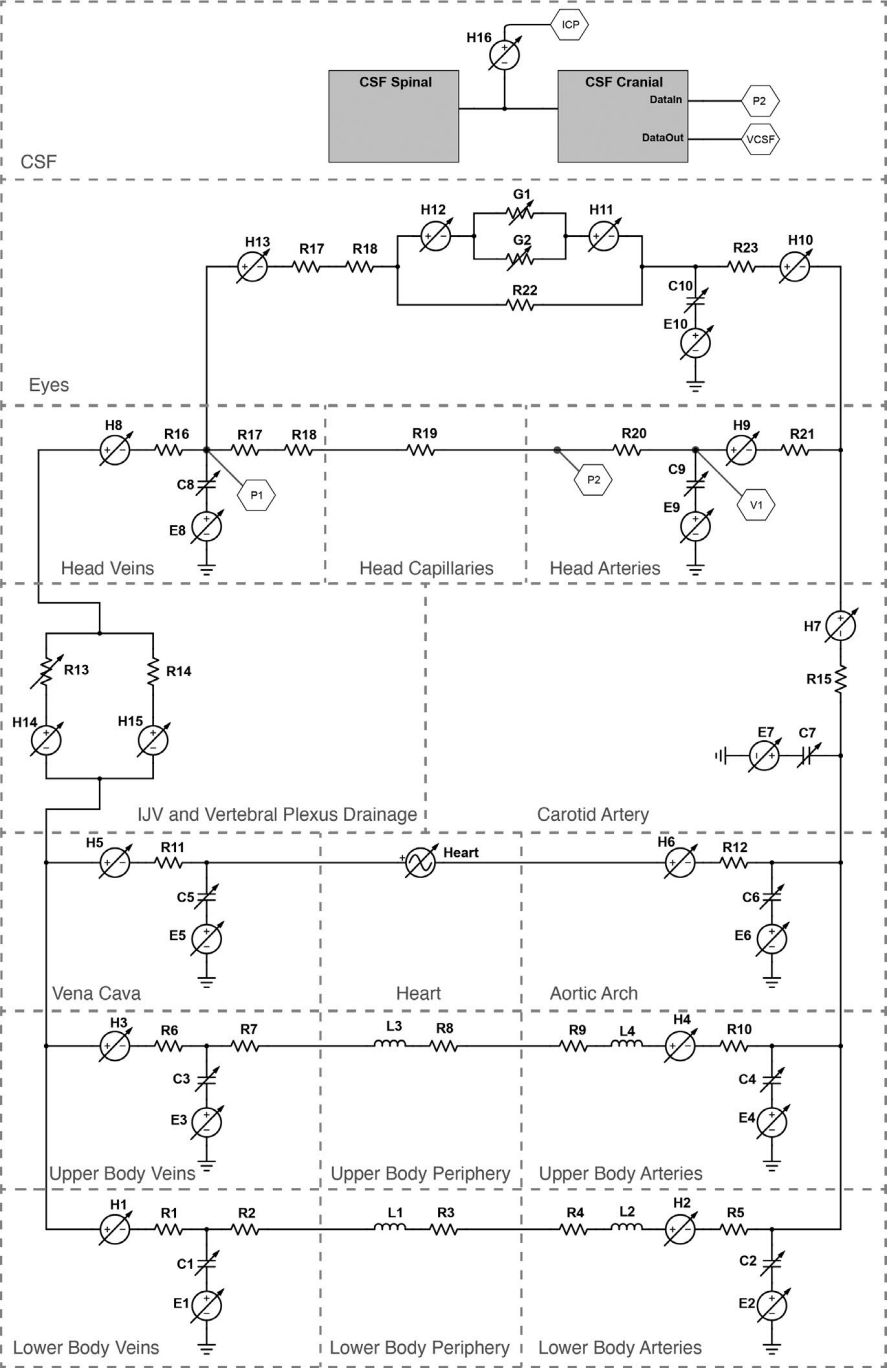
Circuit representation of the 18‐compartment numerical model. Reference Table 1 for circuit labels. The aqueous humor fluid system is contained within the eye compartment. There is no fluid exchange between the CSF spaces and the rest of the fluid system, however, important data parameters are exchanged between the two systems and this influences flow behavior. i.e., *C*8 = *f*(*V*1, *VCSF*), *C*10 = *f*(*ICP*) and, *VCSF* = f(*P*2)

The model is a multicompartment‐lumped parameter model composed of three subsystems: the circulatory sub‐model, the CSF sub‐model, and the aqueous humor sub‐model (Figure 1 and Table 1). Overall vessel behavior is described by combinations of four discrete model components representing hydrostatic gradients, vessel compliance, flow resistance, and flow inertia. These components are described in detail in the appendix; access to the full model is also provided there. A key feature of this model is the incorporation of compressive forces exerted on vessels by the weight of tissues and the subsequent release of those forces in microgravity. Incorporation of the effect of tissue weight accounts for their contribution to vessel pressure and volume resulting from transmural pressure changes in microgravity. Tissue weight, calculated from the radius of the neck, chest, and waist and then converted to an equivalent water column, exerts a compressive force on the outside of vessels limiting their ability to expand with increasing internal pressure. Therefore, in microgravity, compliant vessels experience a larger than normal transmural pressure and larger than normal volume. This approach to modeling offers an advantage over ground‐based analogs of microgravity, such as head down tilt (HDT), which is unable to replicate tissue weightlessness and the loss of hydrostatic gradients.

**TABLE 1:** Circuit labels and values for Figure 1. Values are either static or calculated as the simulation is run. The sources are from literature or tuned to create a reasonable output value

Label	Description	Values	Sources
Compliances			
C1	Lower body veins compliance	1.0 e8 m^3^/Pa	Tuning
C2	Lower body arteries compliance	5.0 e−9 m^3^/Pa	Tuning
C3	Upper body veins compliance	6.0e−8 m^3^/Pa	Literature
C4	Upper body arteries compliance	5.0e−9 m^3^/Pa	Literature
C5	Vena Cava compliance	6.0e−8 m^3^/Pa	Literature
C6	Aorta compliance	6.0e−9 m^3^/Pa	Literature
C7	Carotid artery compliance	1.5e−11 m^3^/Pa	Tuning
C8	Head veins compliance	Calculated	N/A
C9	Head arteries compliance	1.0e−11 m^3^/Pa	Literature
C10	Eye compliance	Calculated	N/A
External Pressures			
E1	Lower body veins external pressure	Calculated	N/A
E2	Lower body arteries external pressure	Calculated	N/A
E3	Upper body veins external pressure	Calculated	N/A
E4	Upper body arteries external pressure	Calculated	N/A
E5	Vena Cava external pressure	Calculated	N/A
E6	Aorta external pressure	Calculated	N/A
E7	Carotid artery external pressure	Calculated	N/A
E8	Head veins external pressure	Calculated	N/A
E9	Head arteries external pressure	Calculated	N/A
E10	Eye external pressure	Calculated	N/A
Hydrostatic Pressures			
H1	Lower body veins hydrostatic gradient	Calculated	N/A
H2	Lower body arteries hydrostatic gradient	Calculated	N/A
H3	Upper body veins hydrostatic gradient	Calculated	N/A
H4	Upper body arteries hydrostatic gradient	Calculated	N/A
H5	Vena Cava hydrostatic gradient	Calculated	N/A
H6	Aorta hydrostatic gradient	Calculated	N/A
H7	Carotid artery hydrostatic gradient	Calculated	N/A
H8	Head veins hydrostatic gradient	Calculated	N/A
H9	Head arteries hydrostatic gradient	Calculated	N/A
H10	Hydrostatic gradient from head center to eye center	Calculated	N/A
H11	Hydrostatic gradient from eye center to front of eye	Calculated	N/A
H12	Hydrostatic gradient from front of eye to eye center	Calculated	N/A
H13	Hydrostatic gradient from eye center to head center	Calculated	N/A
H14	Jugular Vein hydrostatic gradient	Calculated	N/A
H15	Vertebral Plexus hydrostatic gradient	Calculated	N/A
H16	CSF hydrostatic gradient	Calculated	N/A
Inertance			
L1	Lower body capillaries inertance	Calculated	N/A
L2	Lower body arteries inertance	Calculated	N/A
L3	Upper body capillaries inertance	Calculated	N/A
L4	Upper body arteries inertance	Calculated	N/A
Vessel Resistances			
R1	Lower body veins resistance	2.0e5 Pa/m^3^/s	Tuning
R2	Lower body venules resistance	6.0e7 Pa/m^3^/s	Tuning
R3	Lower body capillaries resistance	8.0e7 Pa/m^3^/s	Tuning
R4	Lower body arterioles resistance	3.0e8 Pa/m^3^/s	Tuning
R5	Lower body arteries resistance	2.6e7 Pa/m^3^/s	Tuning
R6	Upper body veins resistance	5.0e4 Pa/m^3^/s	Literature
R7	Upper body venules resistance	6.0e7 Pa/m^3^/s	Literature
R8	Upper body capillaries resistance	6.0e7 Pa/m^3^/s	Literature
R9	Upper body arterioles resistance	1.0 e8 Pa/m^3^/s	Literature
R10	Upper body arteries resistance	2.6e7 Pa/m^3^/s	Literature
R11	Vena Cava resistance	Calculated	N/A
R12	Aorta resistance	Calculated	N/A
R13	Jugular Vein resistance	Calculated	N/A
R14	Vertebral Plexus resistance	Calculated	N/A
R15	Carotid artery resistance	Calculated	N/A
R16	Head large veins resistance	1.5e7 Pa/m^3^/s	Tuning
R17	Head small veins resistance	3.1e7 Pa/m^3^/s	Tuning
R18	Head venules resistance	2.9e7 Pa/m^3^/s	Tuning
R19	Head capillaries resistance	2.0e8 Pa/m^3^/s	Tuning
R20	Head arterioles resistance	5.2e8 Pa/m^3^/s	Tuning
R21	Head arteries resistance	1.9e8 Pa/m^3^/s	Tuning
R22	Resistance around the eye	3.0e11 Pa/m^3^/s	Tuning
R23	Resistance to the eye	8.0e10 Pa/m^3^/s	Tuning
G1	Trabecular conductance	0.00029 mL/min	Literature
G2	Uveoscleral conductance	0.0012 mL/min	Literature
Data Nodes			
P1	Fluid pressure at location P1 in Head Veins	Calculated	N/A
P2	Fluid pressure at location P2 in Head Arteries	Calculated	N/A
V1	Fluid volume at location V1 in Head Arteries	Calculated	N/A
VCSF	Cranial CSF fluid volume	Calculated	N/A
ICP	Intracranial fluid pressure	Calculated	N/A

## 2 METHODS

To assess the effects of microgravity (0‐g) and lower body pressure on jugular flow, we simulated the body in the 1‐g supine, 1‐g 6‐degree HDT and in microgravity. For each of these, lower body chamber pressure was simulated at atmospheric pressure (ATM), negative pressure of −40 mmHg (LBNP), or positive pressure of 40 mmHg (LBPP) for a total of nine simulated conditions. In addition to jugular flow, simulated results for central venous pressure (CVP), intracranial pressure (ICP), and intraocular pressure (IOP) for 1‐g supine, 1‐g prone, and microgravity were also reported to compare with experimental measurements made in weightlessness.

The model was initiated in the 1‐g supine position as the baseline condition. Data to initialize were derived from experimental subjects placed in the supine position. Measures on 16 subjects were taken in the laboratory (height, heart height, chest circumference, waist circumference, neck circumference, heart rate, systolic pressure) and on 14 subjects in an MRI (head axial length, CSF length, head artery length, cranium volume, brain volume, CSF volume, eye axial length, carotid artery cross‐sectional area, left jugular vein cross‐sectional area) and are summarized in the model documentation. Fluid volume was established using systolic pressure, cranium volume, brain volume, head CSF volume, carotid cross‐sectional area, and jugular vein cross‐sectional area as initial conditions. The model parameters were tuned such that the model output matched experimentally measured IOP, diastolic pressure, carotid flow rate, and jugular flow rate in the supine position with atmospheric chamber pressure. Confidence in the model was established by validating model outputs against experimental results for supine LBPP, supine LBNP, prone ATM, prone LBPP, and prone LBNP. Once the fluid volume was determined, it was held constant and the pulsatility of the heart was initiated. From here, the model simulated each condition sequentially, allowing the solution to stabilize before transitioning to the next condition. Changing from supine ATM to supine LBNP was simulated by holding body orientation constant and linearly ramping lower body chamber pressure to −40 mmHg. Once supine LBNP stabilized, supine LBPP was simulated by linearly ramping lower body chamber pressure to +40 mmHg. After stabilizing, 6‐degree HDT with atmospheric chamber pressure was simulated by linearly ramping body orientation and chamber pressure from ‐π/2 to −1.675 radians and from +40 to 0 mmHg, respectively. Zero gravity was propagated through the model as a loss of hydrostatic gradients and a change in transmural vessel pressure due to reduced tissue compression. We continued in this fashion until all nine conditions were simulated. Because of the pulsatility of the heart, the average value for each condition (excluding the transition phase) was calculated and used for the data analysis in the results.

To assess the effect of tissue weightlessness, the model was run again in the manner described previously with the tissue pressure factor disabled. To disable the effects of tissue weight, the first term in the external vascular pressure equation (Equation 1) was attenuated to 0.1% of its normal value. Doing this, rather than setting it to zero, maintained model stability and allowed a solution to converge. The external vascular pressure ($P_{\mathrm{ext}}$) equation is:

(1) $$P_{\mathrm{ext}}=r\frac{g}{1G}\left|\sin\theta \right|+P_{\mathrm{chamber}}$$

where *r* is radius of body part, *g* is acceleration caused by gravity, *G* is acceleration caused by gravity on Earth, θ is body orientation, and $P_{\mathrm{chamber}}$ is chamber pressure applied to the lower body.

To understand the contribution of body weight to IJV flow suppression, we ran the model again with lighter than average and heavier than average body parameters. Body parameters are modeled as the circumferences of the neck, chest, and waist. The average body size used in the model was 36 cm, 97 cm, and 83 cm for the neck, chest, and waist respectively. These values were obtained from anthropometric measurements of 16 subjects who contributed experimentally determined measurements for parameters in the model. Light weight and heavy weight individuals were created by decreasing or increasing the three body circumference measurements by 25% before running the model.

## 3 RESULTS

**FIGURE 2:**
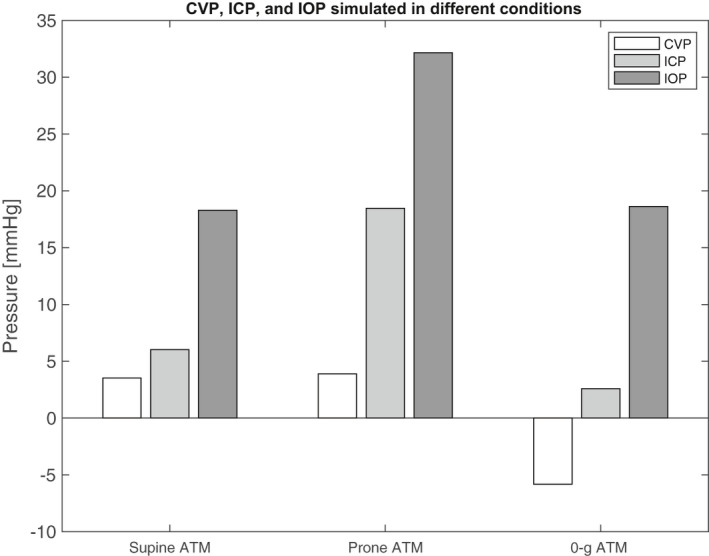
Numerical model results for ICP, IOP, and CVP for multiple environmental conditions

Application of the model to spaceflight conditions predicted the reductions in venous pressure seen with weightlessness (Figure 2). Experimental measurements taken of CVP, ICP, and IOP are reflected qualitatively by the model results. (Buckey, 2006) recorded a 7.6 mmHg reduction of CVP in microgravity relative to preflight supine levels. The model simulated a 9.8 mmHg reduction. (Lawley et al., 2017) recorded an average ICP reduction of 3.8 mmHg (+/‐ 2.9 mmHg) from 1‐g supine values to 0‐g values. The model simulated a 3.5 mmHg reduction. For IOP, (Anderson et al., 2016) hypothesized and experimentally verified that IOP values in microgravity lie above supine values, but below prone values, obtaining values of 16.3 mmHg, 13.7 mmHg, and 20.3 mmHg respectively. The model reflects their qualitative results, simulating microgravity IOP to be above supine and below prone, with values 19.3 mmHg, 18.3 mmHg, and 32.1 mmHg, respectively.

**FIGURE 3:**
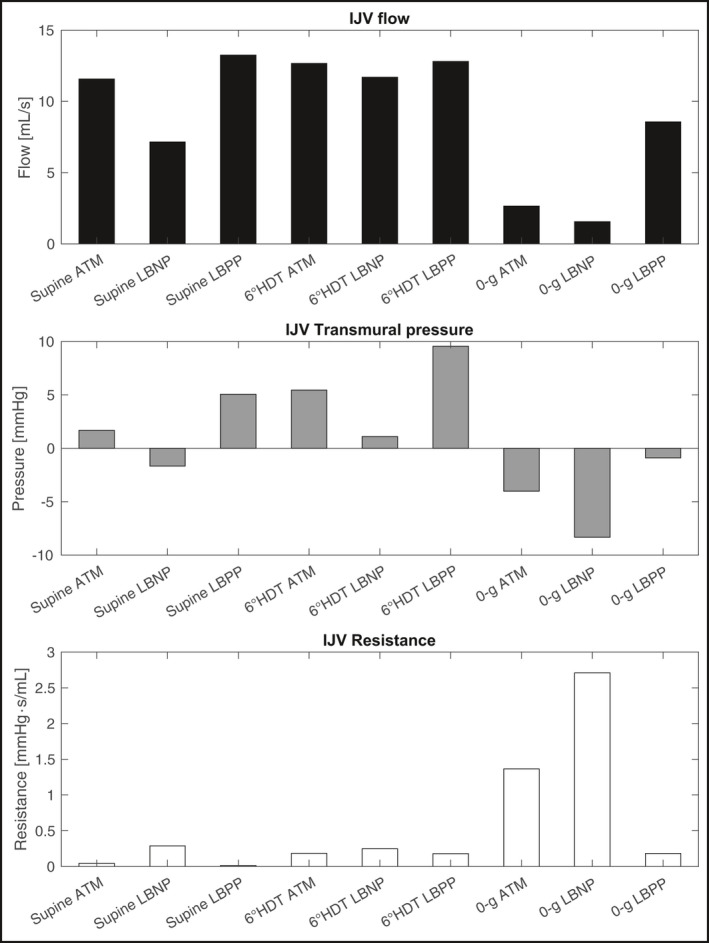
Numerical model results of IJV flow rate, IJV transmural pressure, and IJV flow resistance for multiple environmental conditions

Modeled IJV blood flow in microgravity (2.65 mL/s) was reduced below supine levels (11.57 mL/s) (Figure 3). Venous pressure in the IJV in microgravity (− 4.02 mmHg) decreased compared with the supine value (3.79 mmHg). The reduction in venous pressure within the IJV exceeded the reduction in external IJV pressure from the loss of tissue forces, and so transmural pressure, $P_{T}$, decreased. This is shown by:

(2) $$P_{T}=\frac{P_{IJV\;inlet}+P_{IJV\;outlet}}{2}‐P_{\mathrm{ext}}$$

where $P_{IJV\;inlet}$ is the IJV inlet pressure, $P_{IJV\;outlet}$ is the IJV outlet pressure, and $P_{\mathrm{ext}}$ is the IJV external pressure. Because veins have compliance, this reduction in transmural pressure leads to a reduction in flow area and vessel volume. Figure 3 shows the transmural pressure, IJV flow resistance, and IJV flow rate for a variety of simulated conditions. When transmural pressure is negative, the segments of the IJV with the lowest transmural pressure can collapse, limiting blood flow and leading to the increased IJV resistance observed in the supine LBNP condition and three microgravity conditions.

We also ran the model with a transition from the supine to HDT positions. In this case, we observed that HDT (12.67 mL/s) did not replicate the reduced jugular venous flow seen in microgravity; rather jugular venous flow increased due to the increased vessel blood pressure (7.54 mmHg) in the IJV.

In microgravity, LBNP application further reduced IJV blood flow. These results suggest LBNP may not be a good countermeasure to prevent venous thromboses caused by reduced venous blood flow. Lower body positive pressure (LBPP), however, might be useful. The model predicts that 40 mmHg of LBPP in microgravity may increase IJV flow (2.7 mL/s ATM, 1.5 mL/s with LBNP, 8.7 mL/s LBPP).

**FIGURE 4:**
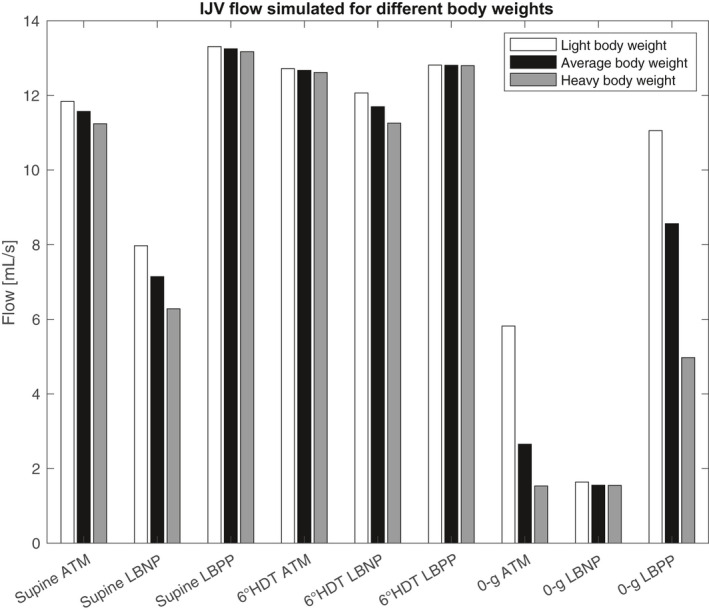
IJV flow simulated for a light, normal, and heavy person for multiple environmental conditions

To determine the source of the reduced venous pressure, the model was run without the effects of tissue weight incorporated. In this configuration, IJV flow stayed mostly the same from supine atmospheric to microgravity atmospheric (see Table 2). Additionally, CVP, ICP, and IOP trends showed worse alignment with the experimentally measured values of those variables from (Buckey et al., 2006), (Lawley et al., 2017), and (Anderson et al., 2016). ICP in particular increased from the supine to microgravity conditions rather than decreasing to match experimental values. These results show that inclusion of tissue weight is critical to correctly simulating microgravity effects on IJV flow, as well as on CVP, ICP, and IOP. This is logical because this model was specifically developed to incorporate the effects of tissue weight. Importantly, however, the model was not tuned to produce particular microgravity results. The model was tuned using the experimental results from 1‐g studies, and the microgravity results were extrapolated using the model. Another set of simulations were run on a light weight and heavy weight individual to further investigate the relationship tissue weight has on the magnitude of microgravity‐induced changes. Figure 4 shows the resulting IJV flow. The IJV flow in the light weight individual was suppressed less by the microgravity environment (51% flow reduction) than the IJV flow of the heavy weight individual (86% flow reduction).

**TABLE 2:** CVP, ICP, IOP, and IJV flow simulated with and without tissue weight effects.

	CVP	CVP no tissue weight	ICP	ICP no tissue weight	IOP	IOP no tissue weight	IJV flow	IJV flow no tissue weight
units	mmHg	mmHg	mmHg	mmHg	mmHg	mmHg	mL/s	mL/s
Supine ATM	3.53	3.53	6.03	−6.27	18.28	18.06	11.57	12.36
0‐g ATM	−5.82	3.49	2.58	0.00	18.61	25.28	2.65	12.38
Difference	−9.35	−0.03	−3.44	6.27	0.33	7.22	−8.92	0.02

## 4 DISCUSSION

The model predicts reduced jugular vein flow in microgravity, agreeing with IJV observations made in spaceflight. The reduced flow result is caused by a drop in IJV transmural pressure leading to a narrowed jugular vein cross section, which increases flow resistance through the IJV. The crosssection and therefore resistance of the IJV depends on the transmural pressure: positive transmural pressure opens the IJV and, conversely, a negative transmural pressure narrows it. In the model, transmural pressure is low across the IJV because venous pressure throughout the cardiovascular system is reduced in microgravity (Buckey et al., 2001). The vertebral venous plexus, which does not have compliant walls, does not change its resistance, leading to a diversion of flow from the IJV to the vertebral plexus.

This result agrees well with the experimental measurements of venous pressure, IJV flow, and IJV cross‐sectional area from spaceflight. Both central venous pressure and peripheral venous pressure have been measured directly in space and fall below supine values (Buckey et al., 1996, 2001; Kirsch et al., 1984). Although these direct measurements were made early in spaceflights, little evidence exists to suggest that venous pressures increase with time in space. Over time, spaceflight leads to an approximate 11% total blood volume reduction, which would serve to reduce venous pressures further (Buckey, 2006). Marshall‐Goebel et al. measured IJV cross‐sectional area preflight, and on days 50 and 150 of spaceflights on the ISS. In their study, IJV cross‐sectional area decreased from 80.7 mm^2^ supine to 70.3 mm^2^ at day 50 of spaceflight (a 13% reduction from supine), and 60 mm^2^ at day 150 of spaceflight (a 26% reduction from supine). Stagnant and retrograde IJV flow was noted at both day 50 and day 150 of spaceflight.

The model IJV pressure results are inconsistent, however, with the non‐invasive IJV pressures measured in the Marshall‐Goebel et al. study. In their study, non‐invasive IJV pressure increased from 17.3 mmHg supine to 21.1 at day 50 but decreased to 15.8 at day 150. This non‐invasive technique used was likely providing overestimates of pressure (as the authors note). Also, the higher pressures at day 50 would be inconsistent with the reduced cross‐sectional areas and flows seen (i.e. increased IJV pressure would likely lead to increased flow). The most likely explanation is that IJV internal pressure was below supine values despite the reported non‐invasive values.

Some parabolic flight studies show increases in IJV volume immediately upon entering weightlessness compared with supine (Lawley et al., 2017), although this is not always noted (Lee et al., 2020). The model results show narrowing of the IJV in 0‐g relative to 1‐g supine. If further experimentation shows definitively that IJV area is increased in acute weightlessness exposure, the disagreement of the model with experimental results might be a result of how the acute fluid shift is modeled. With acute initial exposure to 0‐g, venous blood volume above the heart may be elevated more than is accounted for in the model leading to an increase in IJV volume above supine values. With continued microgravity exposure, overall blood volume is reduced leading to venous volume above the heart to eventually settle below supine levels, which may explain how IJV area could be increased acutely, but settle below supine values later in the flight.

In the HDT simulation, IJV flow increased relative to supine. This result is consistent with experimental measurements of acute exposure to HDT (Lawley et al., 2017). IJV flow is increased in HDT relative to supine because the venous pressures are elevated in HDT rather than decreased as they are in microgravity.

The model findings suggest that LBNP may not be a useful countermeasure for increasing IJV flow volume. The model showed that LBNP further reduced IJV pressure, further increased flow resistance, and further diminished the suppressed IJV flow in microgravity relative to 1‐g supine flow conditions. This is supported by the data from the Marshall‐Goebel et al. study, which shows a further reduction in IJV cross‐sectional area with LBNP as well as mixed results in the efficacy of LBNP to improve continuity of IJV flow. Lower body positive pressure, however, might be useful. Data from the model predict that LBPP would increase IJV flow volume in space. This is a modeling result, however, and needs to be tested in spaceflight. Documenting consistently improved flow continuity with LBPP application will be vital to assessing whether it has promise as a countermeasure.

From the light and heavy body weight simulation, the model suggests that body weight is a predictive indicator of the severity of IJV flow suppression. Interestingly, body weight has been shown to correlate with manifestation of spaceflight‐associated neuro‐ocular syndrome (SANS) symptoms (Buckey et al., 2018).

### 4.1 Limitations

There is no baroreflex system in the model. However, given the environmental conditions—gravity, body orientation, or chamber pressure—the model is provided with experimentally determined heart rate and systolic pressure matching those conditions. Thus, future work should incorporate these control mechanisms to improve model fidelity and further validate the predicted findings, in particular the notion that LBPP may be a useful countermeasure to facilitate IJV flow.

We reported on and analyzed mean blood flow for each condition, and the effects of blood pulsatility on IJV thrombosis were not considered. Our conclusions were based solely on blood flow volume. It is possible that flow dynamics may play an important role in thrombotic risk. A future study including the effects of blood pulsatility or blood flow dynamics in addition to flow volume would provide a more complete analysis.

The model does not simulate physiological changes in the cardiovascular system with long‐term microgravity exposure nor does it simulate transfer of fluid between the intravascular space and the extravascular space, with the exception of creating and filtering the aqueous humor in the eye. Rather, it represents a quasi‐steady‐state condition after an initial alteration, but cannot simulate effects on long timescales. Although this limitation exists, in this paper we have extrapolated the acute change of reduced systemic venous pressures predicted by the model to the long‐term effects of spaceflight on jugular venous flow. We have done this because there is little evidence suggesting that venous pressures will increase gradually after the initial acute effect. In fact, documented blood volume reduction associated with long‐term spaceflight would likely lead to further reductions in venous pressures. Therefore, this paper ventures to propose a hypothesis for jugular venous flow reduction based on systemic reductions of venous pressures.

The effects of intrathoracic pressure and breathing on decreased CVP in microgravity relative to a supine baseline, as described by Videbaek et al, is not simulated in this model (Videbaek & Norsk, 1997). This is likely not a significant omission. A major reduction in intrathoracic pressure in space would be accompanied by a significant increase in lung volume (in addition to effects on CVP). Lung volume in microgravity is only slightly increased compared with the supine position on Earth, suggesting that there is not a major change in intrathoracic pressure in space (Buckey et al., 2001; Elliott et al., 1994; West & Prisk, 1999). This is one reason why the reductions in central venous pressure in space are more likely to be related to the loss of tissue weight than to an effect on intrathoracic pressure (Buckey et al., 2001). If, however, intrathoracic pressures were included this would likely strengthen the findings in this paper as this would serve to further reduce venous pressures.

Use of a lumped‐parameter modeling approach greatly simplifies physiological systems, anatomy, and physiological parameters and so may be inaccurate. Nevertheless, the simulated results capture major system dynamics and align with real world results. Therefore, the model can be a valuable tool for forming and testing hypotheses.

## 5 CONCLUSION

We have developed a numerical model of the cardiovascular system capable of reproducing hemodynamic responses to gravitational change, body orientation, and external chamber pressure on the lower body. Most importantly, the model integrates the effects of tissue compressive forces on the effective compliance of vasculature. The simulated responses compare well with experimental microgravity data published in literature, and we used it to generate a new hypothesis for the mechanism of reduced jugular venous flow in microgravity.

## AUTHOR CONTRIBUTION

M.L. performed model output analysis and interpretation and was primarily responsible for writing the manuscript. S.P. oversaw and was involved in the design and development of the model, as well as the model output analysis. V.A. was also involved in model design, development, and output analysis. A.C. was responsible for model design and development and assisted in model output analysis. R.L. conducted and documented the model sensitivity analysis. A.A. collected measurement data from volunteers and assisted in the interpretation of the model outputs. K.M. collected measurement data from volunteers. A.F. oversaw volunteer measurement data collection and managed volunteer recruitment. R.H. assisted in the manuscript development and the model interpretation. J.B. was the principal investigator and was involved in the model design and development, volunteer measurement collection, model sensitivity analysis, model output analysis and interpretation, and manuscript writing. All authors assisted with revising the final work and approved the final version to be published. All authors agree to be accountable for all aspects of the work and ensuring that questions about the accuracy or integrity of any part of the work are appropriately investigated and resolved.

## 1 LINK TO THE MODEL

This lumped‐parameter model is available at: http://weightless.dartmouth.edu/

## 2 THE CIRCULATORY SUB‐MODEL

The circulatory sub‐model consists of the heart, a simplified body loop, and a simplified head loop as shown in Figure 1. The heart is modeled as a pressure driven flow source with a sine wave input to simulate the waveform of a heartbeat. The heart's output is provided by experimentally determined heart rates and systolic pressures. The simplified body loop consists of upper and lower body arteries, upper and lower body capillary systems, upper and lower body veins, and the inferior vena cava. The simplified head loop consists of carotid arteries, head arteries, brain capillaries, head veins, jugular veins, and the vertebral venous plexus. The model takes care to properly simulate the split cranial drainage pathway through the jugular vein and vertebral plexus. For example, with low internal jugular pressures, such as would occur with standing, the jugular vein collapses, shunting blood through the vertebral plexus.

## 3 THE CSF SUB‐MODEL

The CSF sub‐model consists of the cranial CSF volume and spinal CSF volume shown at the top of Figure 1. The CSF pulsatile flow is caused by the head arteries’ pressure oscillations, which push on the compliant cranial CSF volume. As the pressure in the cranium oscillates, the overall fluid volume within the cranium must remain constant. This constraint is enforced in the model. The cranial CSF volume, head arterial volume, and head venous volumes act as three separate balloons (compliant reservoirs) inside the cranium; when the volume of one balloon increases, the others vary according to the pressure‐volume‐flow balance. The spinal CSF volume provides an overflow mechanism for the cranial CSF volume. The pulsatile flow between the cranial and spinal CSF spaces is on the order of 100–200 mL/min and the normal adult produces between 400 and 60 mL of CSF per day (Sakka et al., 2011). Because flow generated by CSF production is less than 0.5% of CSF pulsatile flow, the model is simplified by setting CSF production and drainage to zero. The CSF sub‐system and circulatory sub‐system interact via pressure‐compliance connections and volume constraints only, influencing the head veins drainage compliance and the eye compliance.

## 4 THE AQUEOUS HUMOR SUB‐MODEL

The aqueous humor sub‐model simulates the blood flow and pressure distribution related to aqueous humor formation. Aqueous humor flow, and specifically its production and drainage, is an important consideration in the model because it is a variable that determines IOP per the Davson Equation:

(1) $$P_{\mathrm{IOP}}=P_{\mathrm{EVP}}+F_{\mathrm{AH}}\times R_{\mathrm{AH}}$$

where $P_{\mathrm{IOP}}$ is intraocular pressure, $P_{\mathrm{EVP}}$ is episcleral venous pressure, $F_{\mathrm{AH}}$ is formation rate of aqueous humor, and $R_{\mathrm{AH}}$ is the outflow resistance of aqueous humor. The sub‐model consists of four resistance elements, four gravity elements, one compliance element, and two drainage terms. Two of the resistances, “AH loop” and “Eye vasculature,” tune the flow of blood that is filtered to produce aqueous humor. The other two define the eye venule and eye vein flow resistance. The four gravity terms allow for simulations of IOP at the center of the eye and at the front of the eye where IOP is experimentally measured. Two drainage terms represent the trabecular meshwork and the uveoscleral drainage pathways for aqueous humor. The compliance element defines the compliant eye volume.

## 5 SUB‐MODEL INTERACTIONS

The CSF, circulatory, and aqueous humor sub‐systems interact with each other through shared pressure boundaries and through indirect fluid transfer mechanisms. The sub‐models act as a coherent representation of the body's fluid mechanical process via a single “working fluid.” The model exchanges this “working fluid” between the sub‐models by passing the fluid through flow restrictions that represent the creation/destruction of one fluid and the destruction/creation of another (e.g., blood in the ocular arteries being converted into aqueous humor in the ciliary body and being drained back to the venous system in the trabecular meshwork and uveoscleral pathways). This simplification greatly stabilized the model while maintaining conservation of mass.

## 6 MODEL COMPONENTS

We defined four vascular elements to model orientation and gravity‐dependent changes in the numerical model: A Hydrostatic Gradient Element, Compliance Element, Resistance Element, and Inertia Element. We applied these components to each of the fluid flow conduits within the system model (e.g., head arteries, venous plexus) which enabled us to specifically model how the different vascular systems responded to varying flows, pressures, posture, and gravity.

## 7 HYDROSTATIC GRADIENT ELEMENT

A custom hydrostatic gradient term captures the effects of changing gravity orientation and magnitude on flow systems with flow loops and branch points. The hydrostatic gradient term allows a user to define the orientation of the vessel with respect to the body's vertical axis and the length of the pipe segment. The vessel orientation and length are in one dimension, defined as the angle out‐of‐plane from the body. The magnitude of gravity and the orientation of the body with respect to gravity are external inputs to the component, which allows these variables to be changed in all models in the system by changing one, top‐level global input. We define each pipe orientation relative to the body such that changing positions impacts flow resistances and pressures in each vessel. The pressure gradient caused by the force of gravity is defined by Equation 2:

(2) ΔP=ρgLcosθ

where ΔP is pressure change between vessel inlet and outlet, *ρ* is fluid density, g is acceleration caused by gravity, L is length of the vessel, and *θ* is angle between the gravity vector and the vessel.

## 8 COMPLIANCE ELEMENT

A custom compliance element captures the vessel wall elasticity. In other contexts, compliance sometimes refers to fluid compressibility, but in the context of this model it describes the change in volume in a tube or chamber with flexible walls.

Compressive forces exerted by the weight of tissue counters the vessel's internal pressure, in effect reducing the vessel's ability to expand under increased internal pressure. Our compliance element changes volume according to transmural pressure defined in Equation 3:

(3) $$C=\frac{\mathrm{dV}}{\mathrm{dP}}$$

where *C* is compliance, and $\frac{\mathrm{dV}}{\mathrm{dP}}$ is change in volume caused by a change in transmural pressure.

Incorporating this information into each vessel allows the model to calculate local transmural pressure and adjust local fluid volume accordingly.

Anthropometric information determines external pressure on the vessel. The radius of each body part is converted to an equivalent water column of external pressure which acts on the appropriate vascular vessels as the tissue weight. Anthropometric dimensions used in this model are the neck, chest, and waist circumferences. Localized pressures (LBNP/LBPP) are added to the lower body tissue external pressure term to further influence vessel behavior. External vascular pressure is calculated by:

(4) $$P_{\mathrm{ext}}=r\frac{g}{1G}\left|\sin\theta \right|+P_{\mathrm{chamber}}$$

where $P_{\mathrm{ext}}$ is external vascular pressure, *r* is radius of the body part where the measurement taken, g is acceleration caused by gravity, *G* is acceleration caused by gravity on Earth, *θ* is body orientation, and $P_{\mathrm{chamber}}$ is chamber pressure applied to the lower body (i.e. LBNP).

## 9 RESISTANCE ELEMENT

The built‐in Simscape™ resistance element models flow resistance through vessels according to Equation 5:

(5) $$R=\frac{\Delta P}{Q}$$

where, R is resistance to flow, ΔP is pressure drop, and *Q* is flow rate. The pressure drop in a vessel is calculated as a function of the vessel's flow rate, Reynold's number, diameter, and length.

The diameter and length are specified by the user input using anthropometric information and measured nominal diameter of the fluid conduit when it is available. When anthropometric information is not available for the fluid conduit, diameter and length are based on literature values and reasonable biological estimates.

## 10 INERTIA ELEMENT

The built‐in Simscape™ inertia element models inertia through vessels according to Equation 6 and 7:

(6) $$I=\frac{\Delta P}{\frac{\mathrm{dQ}}{\mathrm{dt}}}$$

(7) $$I=\rho \frac{L}{A}$$

where, *I* is inertance, ΔP is pressure drop, $\frac{\mathrm{dQ}}{\mathrm{dt}}$ is change in flow rate per change in time, *L* is effective vessel or path length, *A* is flow cross‐sectional area and, *ρ* is fluid density.

No user input is required for the inertia term. Inertia terms are not used in all fluid conduits in the system model because inertia is often negligible, particularly on the venous side of the circulatory system. The Simscape™ element requires a length and area to determine the inertance. We derive non‐physical length and areas to input into this block based on literature values of arterial inertance.

## 11 IJV AND VP FLOW BEHAVIOR

The jugular vein cross‐sectional area imitates a binary switch, fully open when under positive transmural pressure conditions and closed in negative transmural pressure conditions. For the model solver to run smoothly, the cross‐sectional area ranges from 1/100 of its unstressed area to its fully unstressed area. A steep ramp is used between open and closed to smooth out the transition between the open and closed conditions. This transition occurs between −2 and 0‐mmHg transmural pressure. Any flow that does not pass through the IJV passes through the VP. Fluid volume is conserved.

## 12 MODEL PARAMETERS AND TUNING

Model parameters used have been experimentally determined, extracted from literature, or estimated through the process of tuning the model. Final parameter values did not strictly use the values as reported in the literature or as measured from experiments, but when it deviated, it matched the appropriate order of magnitude. The final parameter values were determined by tuning the model to match the IOP, diastolic pressure, carotid flow rate, jugular flow rate, and overall pressure distributions determined experimentally for the body in 1‐g supine ATM conditions. Systolic pressure was an input to the model. Experimentally determined measurements were gathered from 16 subjects in the laboratory and 14 subjects in an MRI. They are summarized in Tables 3 and 4.

**TABLE 3:** System level model input values based on guidance from literature. Model inputs without literature references were determined by model tuning. Model inputs values used in the model that differ from the listed literature value matched the order of magnitude of the literature value

	Values Used in Model	Literature Values	Literature References
Aorta Parameters			
Angle with Body	0 rad	—	—
Compliance	6.0e−9 m^3^/Pa	2.8e−9 m^3^/Pa 1.1e−8 m^3^/Pa	(Conlon et al., 2006) (Lakin et al., 2003)
Diameter	3.2 cm	3.11 cm, females 3.36 cm, males	(Mao et al., 2008)
Upper Body Arteries Parameters			
Angle with Body	π rad	—	—
Compliance	5.0 e−9 m^3^/Pa	8.1e−10 m^3^/Pa 7.5e−9 – 1.5e−8 m^3^/Pa	(Conlon et al., 2006) (Lakin et al., 2003)
Initial Volume	400 cm^3^	400 cm^3^ (half of arteries and arterioles)	(Conlon et al., 2006)
Resistance	2.6e7 Pa/m^3^/s	1.3e7 Pa/m^3^/s	(Conlon et al., 2006)
Arterioles Resistance	1.0 e8 Pa/m^3^/s	3.6e7 Pa/m^3^/s	(Conlon et al., 2006)
Lower Body Arteries Parameters			
Angle with Body	π rad	—	—
Compliance	5.0 e−9 m^3^/Pa	—	—
Initial Volume	400 cm^3^	—	—
Resistance	2.6e7 Pa/m^3^/s	—	—
Arterioles Resistance	3.0e8 Pa/m^3^/s	—	—
Body Capillaries Parameters			
Upper Body Capillaries Resistance	6.0e7 Pa/m^3^/s	2.7e7 Pa/m^3^/s	(Conlon et al., 2006)
Lower Body Capillaries Resistance	8.0e7 Pa/m^3^/s	—	—
Body Veins Parameters			
Angle with Body	0 rad	—	—
Compliance	6.0e−8 m^3^/Pa	3.8e−7 – 1.5e−6 m^3^/Pa	(Lakin et al., 2003)
Initial Volume	1475 cm^3^	1450 cm^3^ (half of veins, venules, and capillaries)	(Conlon et al., 2006)
Resistance	5.0e4 Pa/m^3^/s	3.2e6 Pa/m^3^/s	(Conlon et al., 2006)
Venules Resistance	6.0e7 Pa/m^3^/s	1.2e7 Pa/m^3^/s	(Conlon et al., 2006)
Lower Body Veins Parameters			
Angle with Body	0 rad	—	—
Compliance	1.0 e8 Pa/m^3^/s	—	—
Initial Volume	1475 cm^3^	—	—
Resistance	2.0e5 Pa/m^3^/s	—	—
Venules Resistance	6.0e7 Pa/m^3^/s	—	—
Vena Cava Parameters		—	—
Angle with Body	0 rad	—	—
Compliance	6.0e−8 m^3^/Pa	1.3e−7 m^3^/Pa 3.8e−7 m^3^/Pa	(Conlon et al., 2006) (Lakin et al., 2003)
Diameter	3 cm	3 cm	(Conlon et al., 2006)
Length	50 cm	50 cm	(Conlon et al., 2006)
Hydrostatic Length	20 cm	—	—
Initial Volume	353 cm^3^	500 cm^3^	(Conlon et al., 2006)
Carotid Arteries Parameters			
Angle with Body	0 rad	—	—
Compliance	1.5e−11 m^3^/Pa	—	—
Head Arteries Parameters			
Angle with Body	0 rad	—	—
Arterioles Resistance	5.2e8 Pa/m^3^/s	—	—
Capillaries Resistance	2.0e8 Pa/m^3^/s	—	—
Compliance	1.0e−11 m^3^/Pa	1.1 e−10 m^3^/Pa	(Linninger et al., 2009)
Resistance	1.9e8 Pa/m^3^/s	—	—
Jugular Vein Parameters			
Angle with Body	0 rad	—	—
Vertebral Plexus Parameters			
Diameter	0.55 cm	—	—
Angle with Body	π rad	—	—
Head Veins Parameters			
Angle with Body	π rad	—	—
Large Veins Resistance	1.5e7 Pa/m^3^/s	—	—
Small Veins Resistance	3.1e7 Pa/m^3^/s	—	—
Venules Resistance	2.9e7 Pa/m^3^/s	—	—
CSF Parameters			
Angle with Body	π/2 rad	—	—
Cranial Compliance	1.78e−10 m^3^/Pa	3.8e−10 m^3^/Pa	(Linninger et al., 2009)
Subarachnoidal Space Diameter	1.12 cm	—	—
Subarachnoidal Space Length	20 cm	—	—
Spinal Diameter	1 cm	1 cm	(Loth et al., 2001)
Spinal Length	50 cm	50 cm	(Loth et al., 2001)
Spinal Volume	80 cm^3^	81+/−13 cm^3^	(Edsbagge et al., 2011)
Eye Parameters			
Angle with Body	π/2 rad	—	—
Initial Volume	6.7 cm^3^	—	—
Resistance to the Eye	8.0e10 Pa/m^3^/s	—	—
Resistance around the Eye	3.0e11 Pa/m^3^/s	—	—
Aqueous Humor Parameters			
Trabecular Outflow Facility	0.00029 cm^3^/min	0.00028–0.00030 cm^3^/min	(Selvadurai et al., 2010), Table 2
Normal Uveoscleral Outflow	0.00112 cm^3^/min	0.00164 cm^3^/min AH turnover	(Toris et al., 1999)
Uveoscleral Outflow Change	0.02 mmHg^−1^	0.02 mmHg^−1^	(Johnson, 2000)

**TABLE 4:** Experimentally determined parameters used in the model

Parameter	Value	units	Collection method
Height	169.78	cm	Lab
Heart height	128.03	cm	Lab
Chest circumference	38.31	in	Lab
Waist circumference	32.78	in	Lab
Neck circumference	14.19	in	Lab
Head axial length	15.96	cm	MRI
CSF length (height from eyes to top of head)	10.24	cm	MRI
Head artery hydrostatic length	4.89	cm	MRI
Cranium volume	1406.50	cm^3^	MRI
Brain volume	1201.50	cm^3^	MRI
CSF volume	212.53	cm^3^	MRI
Eye axial length	2.43	cm	MRI
Carotid cross‐sectional area	0.58	cm^2^	MRI
Jugular vein (left) cross‐sectional area	0.88	cm^2^	MRI
HR, Supine, ATM	63.40	beats/min	Lab
HR, Supine, LBPP	65.10	beats/min	Lab
HR, Supine, LBNP	72.10	beats/min	Lab
HR, Prone, ATM	66.20	beats/min	Lab
HR, Prone, LBPP	73.00	beats/min	Lab
HR, Prone, LBNP	73.00	beats/min	Lab
Systolic Pressure, Supine, ATM	126.70	mmHg	Lab
Systolic Pressure, Supine, LBPP	133.30	mmHg	Lab
Systolic Pressure, Supine, LBNP	122.00	mmHg	Lab
Systolic Pressure, Prone, ATM	120.50	mmHg	Lab
Systolic Pressure, Prone, LBPP	129.30	mmHg	Lab
Systolic Pressure, Prone, LBNP	126.20	mmHg	Lab

## 13 LOCAL SENSITIVITY ANALYSIS

A sensitivity analysis was performed on the model to evaluate the validity of model output and behavior. A local sensitivity analysis (LSA) was performed using “one at a time” (OAT) method (Iooss & Lemaître,). This method varies each variable one at a time while fixing the others. Parameter perturbations of +/‐12.5% and +/‐25% were applied to each of the literature and experimental parameters in Table 5. The parameter passed the LSA test if model outputs stayed within physiological ranges and the model does not become unstable.

**TABLE 5:** Sensitivity Analysis Results

Resistances and Compliances			
Parameter Name	Value used in model	Tested	Passed
Aorta Parameters			
Compliance	6.0e−9 m^3^/Pa	+	+
Body Arteries Parameters			
Total Arterial Inertance	2.0 e−3 mmHg⋅s^2^/cm^3^	+	+
Compliance	5.0 e−9 m^3^/Pa	+	+
Resistances	2.6e7 Pa/m^3^/s	+	+
Arterioles Resistance	1.0 e8 Pa/m^3^/s	+	+
Lower Body Arteries Parameters			
Compliance	5.0 e−9 m^3^/Pa	+	+
Resistances	2.6e7 Pa/m^3^/s	+	+
Arterioles Resistance	3.0e8 Pa/m^3^/s	+	+
Body Capillaries Parameters			
Resistances	6.0e7 Pa/m^3^/s	+	+
Lower Body Capillaries Resistance	8.0e7 Pa/m^3^/s	+	+
Body Veins Parameters			
Compliance	6.0e−8 m^3^/Pa	+	+
Resistances	5.0e4 Pa/m^3^/s	+	+
Venules Resistance	6.0e7 Pa/m^3^/s	+	+
Lower Body Veins Parameters			
Compliance	1.0 e8 Pa/m^3^/s	+	+
Resistances	2.0e5 Pa/m^3^/s	+	+
Venules Resistance	6.0e7 Pa/m^3^/s	+	+
Vena Cava Parameters			
Compliance	6.0e−8 m^3^/Pa	+	+
Carotid Arteries Parameters			
Compliance	1.5e−11 m^3^/Pa	+	+
Head Arteries Parameters			
Arterioles Resistance	5.2e8 Pa/m^3^/s	+	+
Capillaries Resistance	2.0e8 Pa/m^3^/s	+	+
Compliance	1.0e−11 m^3^/Pa	+	+
Resistances	1.9e8 Pa/m^3^/s	+	+
Jugular Vein Parameters			
Compliance	1.3e−8 m^3^/Pa	+	+
Vertebral Plexus Parameters			
Compliance	5.0e−9 m^3^/Pa	+	+
Head Veins Parameters			
Large Veins Resistance	1.5e7 Pa/m^3^/s	+	+
Small Veins Resistance	3.1e7 Pa/m^3^/s	+	+
Venules Resistance	2.9e7 Pa/m^3^/s	+	+
CSF Parameters			
Cranial Compliance	1.78e−10 m^3^/Pa	+	+
Eye Parameters			
Resistance to the Eye	8.0e10 Pa/m^3^/s	+	+
Resistance around the Eye	3.0e11 Pa/m^3^/s	+	+
Arterial, venous volumes and vessel dimensions			
Aorta Parameters			
Diameter	3.2 cm	+	+
Body Arteries Parameters			
Initial Volume	400 cm^3^	+	+
Lower Body Arteries Parameters			
Initial Volume	400 cm^3^	+	+
Body Veins Parameters			
Initial Volume	1475 cm^3^	+	+
Lower Body Veins Parameters			
Initial Volume	1475 cm^3^	+	+
Vena Cava Parameters			
Diameter	3 cm	+	+
Length	50 cm	+	+
Hydrostatic Length	20 cm	+	+
Initial Volume	353 cm^3^	+	+
Vertebral Plexus Parameters			
Diameter	0.55 cm	+	+
CSF Parameters			
Subarachnoidal Space Diameter	1.12 cm	+	+
Subarachnoidal Space Length	20 cm	+	+
Spinal Diameter	1 cm	+	+
Spinal Length	50 cm	+	+
Spinal Volume	80 cm^3^	+	+
Eye Parameters			
Initial Volume	6.7 cm^3^	+	+
Aqueous Humor Parameters			
Outflow Facility	0.00029 cm^3^/min	+	+
Normal Uveoscleral Outflow	0.00164 cm^3^/min	+	+
Uveoscleral Outflow Change	0.02 mmHg^−1^	+	+
Heart			
Heart Rate	various for different body positions, e.g. 63.4 BPM	+	+
Initial Volume	various for different body positions, e.g. 126.7 mmHg	+	+
